# Regulatory tasks of national medical associations - international comparison and the Israeli case

**DOI:** 10.1186/2045-4015-2-8

**Published:** 2013-02-20

**Authors:** Malke Borow, Baruch Levi, Michelle Glekin

**Affiliations:** 1 The Israeli Medical Association (IMA), Ramat Gan, Israel

## Abstract

**Background:**

In many countries, NMAs, along with other stakeholders, play a part in the regulation of physicians. The purpose of this paper is to compare and explain the level of involvement of NMAs in physician regulation in several developed countries, with a specific emphasis on Israel.

**Methods:**

The authors conducted a review of the literature on physician regulation, focusing on licensing and registration, postgraduate training and physician disciplinary measures. Detailed country specific information was also obtained via the websites of relevant NMAs and regulatory bodies and correspondence with select NMAs.

Five test cases were examined in detail: Germany, Israel, the Netherlands, the United Kingdom and the United States. The Israeli case will be discussed at greater length.

**Results:**

Medical licensing usually lies in the hands of the government (on the national or state level). Specialist training, on the other hand, is often self-regulated and entrusted in the hands of the profession, frequently under the direct responsibility of the NMA, as in Israel, the Netherlands and Germany.

In all the countries presented, other than Germany, the NMA is not involved in instituting disciplinary procedures in cases of alleged physician misconduct.

**Discussion:**

The extent to which NMAs fulfill regulatory functions varies greatly from country to country. The relationship between government and the profession in the area of regulation often parallels the dominant mode of governance in the health care system as a whole. Specifically, the level of involvement of the Israeli Medical Association in medical regulation is a result of political, historical and ideological arrangements shaped vis-à-vis the government over the years.

**Conclusions:**

In Continental Europe, co-operation between the NMA and the government is more common than in the USA and the UK. The Israeli regulatory model emerged in a European-like fashion, closer to the Netherlands than to Germany.

The Israeli case, as well as the others, demonstrates the importance of history and ideology in shaping contemporary regulatory models.

## Background

According to the saying, “there is power in numbers”, and nowhere is this more true than in the case of professional associations. What an individual might not be able to achieve, can often be actualized as part of a larger group. Therefore, in a large number of developed and developing countries throughout the world, physicians are organized and/or represented by national medical associations (NMAs).

The role of the NMA is multi-faceted. It may serve as a trade union, professional standards setter, policy maker, ethical arbiter, disciplinarian, or some combination of the above. Certain functions may be statutory, most are not. Although the precise function of the medical association may vary from country to country, there are certain functions that are prevalent in many associations, among them: regulation, quality assurance, CME, ethics and public health policy.

With the development and expansion of the medical world and all that encompasses from an economic, ethical, statutory and policy point of view, most NMAs no longer view themselves solely as trade unions. Some do not see themselves as fulfilling that role at all. In a post by former American Medical Association (AMA) president Peter Carmel, he writes that the British Medical Association (BMA) “calls itself ‘the independent trade union and professional association for doctors and medical students.’ Note the initial emphasis on trade union-certainly something the AMA is not [1].” (*emphasis in the original*)

Even where NMAs do fulfill the traditional role of trade union, their ambit today reaches much further. We set out to explore the key areas in which NMAs are involved. What regulatory functions do they assume? To what extent do they oversee the clinical and quality aspects of the profession? How much are they involved in local or national decision making? And what is the division of responsibility between the profession and the government?

This article focuses on the regulatory functions of NMAs and the balance of power with government in these areas. As these functions have traditionally rested more in the hands of the profession, we have begun here and in further articles will explore NMAs’ involvement in quality improvement and public policy, which in our view are later additions to the definition of professional activity.

Five test cases were examined in detail, describing the actions of the main institutions and organizations in various countries. The Israeli case, which is rarely mentioned in the literature, will be discussed at greater length.

### Regulation and NMAs

The term regulation can be used in many ways and is difficult to define.

The literature on regulation emphasizes the workings, characteristics, failures and merits of regulation through the workings of administrative agencies. These aspects are expressed in one of the most widely cited definitions of regulation, namely as *“sustained and focused control exercised by a public agency over activities that are valued by the community”*[2-4].

Following Levi-Faur and other scholars, this definition can be extended to include not only public agencies, but also business-to-business regulation or civil regulation. This extended definition emphasizes the role of diverse sets of actors and can be used in many fields of policy, including health care. In particular, it allows us to recognize non-governmental entities such as non-profit organizations, associations and labor unions. For purposes of this paper, we shall apply this definition to the field of physician regulation, and examine how administrative agencies monitor traditional tasks of physicians’ professional life. This regulatory action can be broken down into several subsections, including training, licensing and discipline. We shall closely examine the involvement of the NMA in each of the three fields of activity in several countries.

The role of NMAs in training, licensing and disciplinary functions is a complex one. Physicians have long insisted on self-regulation, believing that the complexity of the profession would make regulation by non-professionals ineffective [5]. “From a physician standpoint, it would not be advantageous to have their actions or clinical decisions evaluated by laypeople and members of the public who are not likely to have the necessary training or experience to make those judgments [6].” However, over the years concerns have been raised by the public about trust, transparency, accountability and potential conflicts of interest on the part of physicians [5]. This has led to a shift from viewing self-regulation as a right, to viewing it as a privilege that needs to be fair and transparent in order to earn the public’s trust.

This tension between the legitimate needs of both sides – the public and the experts - is not exclusive to medicine. An ongoing international debate has been taking place in recent years regarding the nature of regulation in the fields of finance, energy, transportation etc. A single, uniformly acceptable regulatory model cannot be found. On the spectrum between no regulation and complete statutory regulation, different models arise, most conspicuously self regulation [7]. This diversity is evident also in the field of physician regulation, where the range and volume of responsibilities assumed by NMAs differs widely, reflecting different modes of governance of national health care systems.

### Theoretical framework

Various sources claim that the history, tradition and particular practices that have developed in each country greatly influence the relationship between the state and the medical profession, including the degree of professional autonomy and the profession’s involvement in regulation [8-11]. These differences pose difficulty in finding a common denominator that would allow for greater generalization among the different countries.

Arguably, functions, roles and fields of responsibility of organizations and institutions are heavily influenced by their political and administrative environment. Saltman, for instance, points to context and culture (themselves shaped by historical, demographic, geographic and political circumstances) as deciding factors in the development of health systems. Even issues such as management, decision making, responsibility and organization of the health system are heavily influenced by these factors [9]. The way NMAs operate similarly reflects the nature, principles and dynamics of their surrounding health care system.

The use of typologies is common in comparative political research in order to frame the institutional context in which policies are developed and applied. Categorization of countries into different clusters according to distinct cultural, historical and organizational attributes of their public systems, has been developed extensively in recent decades, and has served as a useful tool in the hands of researchers aiming to identify, analyze and explicate the logic of policy making and political behavior [12]. A prominent example is Esping-Andersen’s seminal work, in which he distinguishes between three types of welfare regimes in developed countries (liberal, conservative and social-democratic) according to the extent of social entitlements and the individual’s degree of independence from market forces [13]. Similar works in the field of health care and later examinations of Esping-Andersen’s typology paved the way to newer classifications, which differentiated the health care system from other domains of the welfare state. Over the course of time, a widely accepted typology of health care systems has evolved, composed of three major models [12,14-17]:

The National Health Service (or Beveridge) model - characterized by universal coverage, funding from general taxation, and public ownership and/or control of health care delivery. This model is most identified with the UK, as well as Sweden and New Zealand, although all three countries, to varying degrees, have moved away from its pure form.

The social insurance (or Bismarckian) model -compulsory and universal coverage as part of a system of social security. Health care is financed by employer and employee contributions, through non-profit insurance funds, and the provision of health care is through public or private ownership. Germany is a prominent example of this model.

The private insurance model - an employer based or individual purchase of private health insurance. Health care is funded by individual and/or employer contributions and health delivery is predominantly under private ownership. This type is most clearly represented by the US.

As mentioned by Burau and Blank, this typology is characterized by a strong economic orientation that, at least to some extent, marginalizes the social and political dimensions of health care systems. In order to counteract this, other works have introduced new attributes to the definition of health care systems, emphasizing their institutional features [12]. Moran’s work is particularly relevant for our purpose, as he sets out to better account for the institutional characteristics of health care. Moran argues that the emphasis on organizational aspects of health care such as finance and provision captures only one aspect of health care and misses out on other important dimensions. Accordingly, he shifts the focus of analysis from the organization to the governance of health care, *i.e.*, from the economic point of view to the political one: who governs what, and how. Specifically, his analysis relates to the governance of consumption (resource allocation and access to health care services), provision (regulation of hospitals and physicians) and production (regulation of medical innovations) [18,19].

Relying on an institutional-oriented analysis, Moran constructed a model of different types of health care states, which resembles the triangular Beveridge-Bismarck-private insurance model, but emphasizes the political rather than organizational dimensions of governance. Three types appearing in the model are especially relevant for our thesis [18]:

Entrenched command and control health care state –provides extensive public access based on citizenship and extensive control of resource allocation through administrative mechanisms, giving the state a central role in governing the consumption of health care, including hospitals and doctors.

Corporatist health care state – gives greater power to various actors within the health system, as opposed to concentrating power in a central source. This gives statutory bodies such as non-profit insurance funds an important role and limits public control over health care costs. Similarly, the governance of doctors and hospitals is less centralized and they therefore play an important role in the governance of provision.

Market health care state – limits public access to health care. Private hospitals and doctors dominate and remain relatively unchecked.

According to Moran, the governance of doctors is a key factor in our understanding of health care systems, since it involves issues such as market control and regulation of physician’s conduct. Moran details the historical power struggle between the state and the profession over control on the General Medical Council (GMC), one of the central entities of medical regulation in the UK, a struggle that concluded with the “invasion” of state agencies into the traditional self-regulated realm of the medical profession and the resultant weakening of physician autonomy [18].

While the UK is considered a “state centered system”, Germany is a clear example of a corporatist model. In point of fact, the corporatist model guides not only the health care system but also political and economic life in Germany. It is defined by the sharing of decision-making powers among the government, labor unions and employers’ organizations. A deciding factor in corporatism is the delegation of specific governmental rights or activities as provided for by law to other, self-regulating institutions. Within the health care system, physicians’ associations (chambers) are obligated by law to provide health services as defined by the legislature. In turn, the associations and their members have a monopoly over the ambulatory care market– the most prestigious and the most lucrative part of the German health care system [18,20].

The US on the other hand is a classic example of a market-driven health system. Moran refers to the rise of managed care and the commercialization of hospitals and health maintenance organizations (HMOs) during the 1990s as leading forces in the shift to a corporate health care system. The growing corporatization of medicine involves a fundamental change in market arrangements as the rate of solo practitioners sharply declines and the cornerstone of physician economic autonomy– fee for service - erodes accordingly [18].

Moran contributed significantly to the development of health care typology by stressing the political dimension of governance. With regard to medical regulation, he describes the dynamics and the balance of power between the state, the physicians and the market, justifiably concentrating on financial arrangements and control over key regulatory institutions. However, the role of NMAs in the regulation of doctors receives only little attention.

### Hypotheses

Whereas Moran and others focus on the political and economic struggle between the medical profession and the state, we wish to concentrate on the regulatory role played by NMAs. Using governance-oriented classification of health care systems as our theoretical framework, we wish to offer a possible explanation for the variance in the extent of regulatory tasks under NMA responsibility. In order to do so we shall formulate three possible hypotheses regarding the volume of regulatory tasks carried out by NMAs:

1. A deciding factor in corporatism is the delegation of specific governmental rights or activities provided for by law to other, self-regulating institutions. It seems reasonable to expect that in a corporatist model the NMA will be more involved in the routine regulation of physicians than will NMAs in an entrenched command and control health care system, a market health care system or a hybrid model.

2. Following the logic of the preceding assumption, we expect to find that the volume of regulatory tasks carried out by an NMA in a mixed model (of corporatist and one or more additional models) will be lower than the volume of tasks carried out in a solely corporatist model, but higher than in the entrenched command and control health care model or the market health care model.

3. We expect to find little or no difference at all between the entrenched command and control health care model and the market health care model regarding the volume of regulatory tasks carried out by NMAs. In both cases, bodies other than physicians’ associations (the state and the market, respectively) are the key actors in regulating health systems, dominating their financial and organizational aspects (*i.e*. control of resource allocation and public access to health care). Therefore in these cases NMAs are not expected to be extensively involved in physician regulation.

### Methods and case selection

The authors conducted a review of the literature on physician regulation, focusing on the areas of licensing and registration, postgraduate training and specialist certification and physician disciplinary measures. These aspects of medical regulation shall be used in order to examine the scope of involvement of NMAs in medical regulation. We assigned a number between zero and three to each NMA, which reflects the aggregate number of activities led by the NMA. The higher the number, the greater that NMA’s involvement is in physician regulation.

Publications of the World Health Organization Regional Office for Europe and the European Observatory on Health Systems and Policies served as main sources of information regarding medical regulatory mechanisms in different countries. We obtained additional data from journal articles and expert reviews, which were found through the use of search engines such as PubMed and Google-scholar. Key words used: medical associations, professionalism, self-regulation, medical regulation, licensing, registration, specialist training, certification, licensure, disciplinary measures. A similar search regarding the regulatory mechanisms in Israel was conducted in Hebrew. More detailed country specific information was also obtained via the websites of relevant NMAs and regulatory bodies as well as correspondence with select NMAs.

A further five test cases were examined in detail, describing the actions of the main institutions and organizations in the following countries: Germany, Israel, the Netherlands, the United Kingdom and the United States. The Israeli case, which is rarely mentioned in the literature, will be discussed at greater length.

The logic of case selection is based on our theoretical framework. As explained, the USA, the UK and Germany are often described in health policy literature as the archetypes of three different health care regimes. The difference in their method of governance constitutes a potential explanation of the variance in regulatory activity of their NMAs. In contrast, the Dutch health care system is considered a hybrid case, combining both corporatist and state-like elements of governance, and therefore cannot be exclusively categorized to either model [12]. In addition, the Dutch bureaucratic system tends to be less legalistic and more inclined to incorporate pluralistic elements than the German bureaucracy, moving it a little closer to the liberal type of regime. It is also interesting to note that Esping-Andersen himself has labeled the Dutch welfare state “The Dutch Enigma” and stated that “*In any case, the Netherlands remains a Janus-headed welfare regime, combining both social-democratic and conservative attributes*” [21].

In our opinion, this set of cases creates a sound base for comparison which in turn will set the stage for a sequential discussion regarding our fifth and somewhat academically neglected case, Israel.

### Findings

The findings are organized into three sections. Each section covers a major regulatory task: licensing and registration; postgraduate training and specialist certification; physician disciplinary measures.

We begin each section with a general description of the specific task and then move on to examine the division of responsibilities between the profession and the government in the five countries mentioned above. We end each section with a summary of the findings from the reviewed countries.

### Licensing and registration

The licensing and registration of physicians is imperative to ensure that only suitably trained and qualified professionals are able to practice medicine.^a^ In many countries, the responsibility for physician licensing belongs to the Ministry of Health. In contrast, there are only a small number of cases where this responsibility lies with an independent professional organization. Often, however, even where the responsibility lies with the Ministry of Health, there is a role for the medical profession to play as well [22].

In many countries where the medical license is issued by the Ministry of Health, it is issued only following mandatory registration or membership with an independent professional body. Such membership can be a precondition, imposed by law, for authorization to practice medicine. These professional bodies hold the responsibility to maintain a register of licensed physicians in their country.

There are also several countries where physician licensing is the direct or delegated responsibility of an independent professional body, such as Austria, France, Ireland, Latvia and the United Kingdom. In Austria, the Austrian Medical Chamber (*Österreichische Ärztekammer*) was delegated the responsibility for licensing and regulation of physicians by the Ministry of Health, and is accountable to the Minister of Health with regards to granting, amending and withdrawing medical licenses. Similarly, in France, the Minister of Health delegates physician licensing to the professional body: the *Ordre des Médecins*. Medical professionals are required to register with the *Ordre des Médecins* in order to receive authorization to practice.

In other countries, physician licensing is the responsibility of a combination of government, professional and independent bodies. In Spain, the licensing of physicians is carried out by the Ministry of Education and Science and registration is the responsibility of the *Consejo General de Colegios Médicos* (Spanish Medical Association) and the Provincial Councils of the Medical College. Registration with the Medical College is a legal pre-condition to the practice of medicine [22].

There are also differences among countries with regards to the division of responsibility within the country. In some cases, licensing and registration responsibilities lie with a national body, whereas in countries such as the United States and Germany, they are split among the different states.

### Germany

In Germany, medical students undergo more than six years of undergraduate medical education, during which they are required to complete two State licensing examinations and a practical year. The practical year concludes with the second state licensing exam.

The Medical Licensure Act (*Approbationsordnung*), introduced by the Federal Ministry of Health (BMG) in 2003, specifies the education and licensing requirements for doctors in the country, while the 1961 Medical Practitioner’s Act (*Bundesärzteordnung*) is the national law for practicing physicians. These two acts form the legal basis for the practice of medicine in Germany [22,23].

Medical licensing is the responsibility of the Minister of Health of the individual state (*Lander*). The license to practice, issued for an indefinite period following successful completion of the licensing examination, is recognized throughout the country. Reaccreditation or re-licensing is not an official requirement in Germany. Nevertheless the regulation of the State Medical Associations instills a duty on all physicians to complete continued medical education. Furthermore, if doctors do not complete sufficient amounts of CME, they face reduced reimbursement and the withdrawal of their license [24].

Upon receiving the license to practice medicine, doctors must also be registered to practice with the *Ärztekammer* (State Chamber of Physicians) of the Lander in which they wish to work [22]. Physicians must become members of their local chamber of physicians and pay an annual membership fee, which varies in correlation with their earnings. The State Chambers maintain up-to-date registers of their members, with details of their employment and contact information. The German Medical Association (GMA) is the umbrella organization, made up of the 17 State Chambers of Physicians.^b^

### Israel

In Israel, under the 1976 Physicians’ Ordinance, the Ministry of Health is the body responsible for granting the general medical license. After completing 6 years of medical school, graduates must complete a 12 month internship, following which they receive a medical diploma and may then apply for a permanent medical license through the Division of Medical Professions at the Ministry of Health [25]. Israel does not have mandatory re-licensure or re-registration. Physicians who study in Israel are not required to take a licensing exam, as opposed to most foreign educated students.^c^ In addition to 6 years education plus an internship year (and exam, where relevant) the law requires the applicants to be an “upstanding human being” (usually defined as having no criminal record), be an Israeli citizen or permanent resident and have a basic command of the Hebrew language. Registration with the Israeli Medical Association (IMA) is not mandatory; nevertheless, the majority of publicly employed physicians are members of the IMA.

### The Netherlands

In the Netherlands, medical education consists of 6 years undergraduate education. No internship is required for initial registration of physicians, but a residency period is included in specialist training [26]. According to the Individual Healthcare Professions Act (BIG Act) only practitioners who comply with the training and education requirements for their profession may be registered in the BIG-register [27]. Registration is conducted through the Minister of Health, Welfare and Sport, via the BIG-register. The Royal Dutch Medical Association (KNMG) is not involved in the initial registration and licensing of physicians.

While there is no national licensing exam, there are certain conditions doctors must meet before being included in the BIG-register, among them [28]:

♦ They must hold a valid diploma

♦ They must be permitted to practice the profession without restrictions

♦ They must pay the appropriate registration fee

♦ They must not have been placed under supervision due to a psychological disorder

There are two types of medical licenses issued in the Netherlands: a provisional license, whereby professionals are permitted to work under supervision for a two year period before obtaining full registration, and full registration, which allows independent practice for an indefinite period, subject to re-accreditation [22].

Over 350,000 health professionals are registered on the BIG-register, including: dentists, doctors, health care psychologists, midwives, nurses, pharmacists, physiotherapists and psychotherapists. The register includes details of the health professionals’ education, specialist training and any restrictions that have been imposed upon them [27].

### United Kingdom

Medical education in the UK is a 5 or 6 year undergraduate degree, similar to Israel, Germany and the Netherlands but as opposed to the US. Also like Israel and unlike the US, the universities provide their own assessments of graduates for licensing purposes, and there is no national licensing exam.

As of 2009, doctors are required by law to both hold a license to practice and to be registered in order to practice medicine in the United Kingdom. The UK General Medical Council (GMC) is appointed by Parliamentary Act (Medical Act 1983) to conduct the regulation of the medical profession, including licensing and registration [29]. The GMC is a body independent both of the government and of domination by any single group, and is publicly accountable. The Council has 24 members, 12 of whom are doctors and 12 of whom are lay members. Physicians are automatically granted a license to practice upon registration. In order to receive full registration, physicians must submit a Certificate of Experience (completed by their medical school) attesting that their year as an intern was successfully completed. The license to practice is issued for an indefinite period, but beginning in 2012 is subject to requirements of revalidation [30]. The BMA is not involved in the licensing and registration process of doctors in the UK.

### United States

U.S. medical students undergo four years of postgraduate education, after taking a set of prerequisite courses during their undergraduate years. Upon completion, they perform a one year internship, which is the minimum training requirement for obtaining a general license to practice medicine in most states. In order to obtain a medical license, applicants must present proof of their education and training, details of their work history, and pass a medical licensing examination. In addition, they must provide information about their medical history, as well as any arrests and convictions, to their State Medical Board [31].

Each state is responsible for the key functions of physician licensing through its State Medical Board, a statutory agency provided for in the state’s Medical Practice Act.

The governance of State Medical Boards differs across the country. Some boards are independent bodies, and hold all licensing and disciplinary powers. Others operate under a larger umbrella organization such as the Department of Health, and have mixed levels of responsibilities and often an advisory capacity. State Medical Boards are typically made up of physician and non-physician members appointed by the state governor [32].

Doctors may practice in several states, but they must be licensed in each state in which they work. In addition, most states require physicians to periodically re-register after obtaining their initial license.

The Federation of State Medical Boards (FSMB), a non-profit organization, brings some uniformity to the licensing process for the different states. Together with the National Boards of Medical Examiners, the FSMB created and currently administers the United States Medical Licensing Examination. It also provides a Federation Credentials Verification Service which provides verified records of a physician’s core medical credentials [32]. The AMA plays no role in the licensing or registration process.

### Summary-licensing and registration

In both Germany and the United States, medical licensing is the responsibility of the different states as opposed to a national body. In Germany, the general medical license is issued by the State Minister of Health; however, physicians must also be registered with their State Chamber, which is the professional body and part of the GMA. In contrast, in the United States, each State Medical Board is organized differently. Some may be independent bodies, while others are under the control of the Department of Health. The United Kingdom has a system different from the others, with the GMC, an independent body made up of both health professionals and lay persons, holding responsibility for physician licensing.

The situation in the Netherlands is most similar to that of Israel. In both countries the government is responsible for administering the general medical license (Table 1).

**Table 1:** Licensing and registration

**Legislation**	**Responsibility**	**Registration with NMA**	**Licensing requirements**	**Education**	**Licensing exams**	**Re-registration/revalidation**
**GERMANY**						
The Medical Licensure Act (Approbationsordnung) and the 1961 Medical Practitioner’s Act (Bundesärzteordnung)	Government and NMA	Doctors must be registered to practice with the *Ärztekammer* (State Chamber of Physicians) of the Lander in which they wish to work.	License to practice is awarded by the State Minister of Health following the completion of the basic medical qualification which includes one year practical training.	6+ years undergraduate medical education	Yes	Reaccreditation or re-licensing is not an official requirement in Germany. However, State Medical Associations require physicians to complete continued medical education.
**ISRAEL**						
1976 Physicians’ Ordinance	Government	No requirement	Medical education plus 12 month internship, no criminal record, citizenship or permanent resident status, basic command of Hebrew, exams for foreign medical graduates.	6 years undergraduate medical education	Only for foreign medical graduates	Permanent Licenses are issued for an indefinite period. Israel does not have mandatory re-registration or revalidation requirements.
**NETHERLANDS**						
Individual Healthcare Professions Act (BIG Act)	Government	No requirement	Physicians must hold a valid diploma, be permitted to practice the profession without restrictions, pay the appropriate registration fee, and have not been placed under supervision due to a psychological disorder.	6 years undergraduate medical education	No	Permanent licenses are subject to re-accreditation.
**UK**						
Medical Act 1983	GMC	No requirement	Physicians automatically receive a license once they are registered. In order to receive full registration physicians must submit a Certificate of Experience (completed by their medical school) displaying proof that their internship year was successfully completed.	5 or 6 year undergraduate medical education	No	Permanent licenses are subject to revalidation (beginning December 3, 2012).
**US**						
Most states have their own Medical Practice Act.	State Medical Boards	No requirement	Physicians must present proof of education and training, details of their work history, and pass a licensing examination. They must also provide information about their medical history and any arrests or convictions to their State Medical Board.	4 years postgraduate education	Yes	In most states, physicians must periodically re-register and fulfill continuing medical education requirements

### Postgraduate training and specialist certification

Postgraduate medical training is a complex process, and the medical profession places great importance on self-education and assessment, in a way that is transparent and acceptable to the public.

Educational standards and curriculum setting are an integral part of both undergraduate and postgraduate medical education. In some countries the same organization is involved in both of these stages, while in many other countries these roles are separated. Additionally, many countries have bodies that oversee and accredit continuing medical education (CME) initiatives to help ensure that practicing physicians have access to educational resources throughout their careers.

The European Union of Medical Specialties (UEMS) Charter on Training of Medical Specialists in the European Community states that:

*“At a national level, the training of medical specialists is regulated by a National Authority, which can be a combination of competent professional or university organizations, a national board or a national governmental authority****advised by a professional authority***[33]*.”*

### Germany

The German Federal Ministry of Health is responsible for setting uniform standards for undergraduate medical education in the country. Medical education is funded by the respective state governments, which also maintain the medical schools and university hospitals.

Postgraduate training of doctors has been delegated to the medical profession at state level. The German State Chambers of Physicians (*Arztekammern*) are responsible for post-graduate medical training including specialist training, examinations and certification. The Chambers set training standards and administer oral exams. Regulations on the content and design of specialty training are incorporated in state by-laws and the independent statutes of the Chambers. In order to ensure standardization within the country, the GMA has developed federal model training regulations which are followed closely by the individual chambers in their specialized training regulations. Recent reforms in Germany make it mandatory for doctors to hold a specialist degree before they can set up practice within the public health insurance system [23]. Specialty training in Germany usually lasts between 4-6 years, depending on the field [22,23].

### Israel

Israeli medical schools grant the degree of Doctor of Medicine (MD) after six years of study, together with the completion of one year’s rotating internship and the submission of a scientific thesis [34].

Israel has a well-developed system of specialty training. Medical specialization is the jurisdiction of the profession, with the Scientific Council of the Israeli Medical Association (IMA) holding statutory responsibility for both training and specialization procedures of medical specialists, as well as the accreditation of departments for specialty training.

The length of basic post-graduate training in Israel varies from 4 to 7 years, depending on the specialty. Exams are administered in two stages-a stage 1 written exam halfway through training and a stage 2 oral exam during or after the last year of residency.

### The Netherlands

The Royal Dutch Medical Association (KNMG) regulates the training and specialist certification of physicians. The KNMG determines the content of training for medical specialists, the accreditation of training institutes, and the requirements for re-registration of medical specialists [35] via “colleges”, which are regulatory bodies belonging to the KNMG. The rules of the colleges require approval of the Dutch Minister of Health [36].

Specialist training in the Netherlands requires physicians to work as medical residents (doctors in training) in a clinical setting. Residents must be enrolled in the training register of one of three KNMG committees, depending on the field. Training times range from 2-1/2 to 4 years for public health specialists, 3 years for general practitioners and 4-6 years for medical specialists [37].

In order to be registered as a medical specialist, physicians must be registered in the BIG-register as well as one of the training committee, hold an employment contract for the duration of the training and follow a training schedule that corresponds with the training of the college [26].

### United Kingdom

In the United Kingdom, by order of the Medical Act (1983), the GMC regulates all stages of physician training. The GMC is responsible for overseeing the core medical curriculum at the undergraduate level. In April 2010 they began regulating postgraduate training as well, due to a merger between the GMC and the Postgraduate Medical Education and Training Board (PMETB), the body previously responsible for such training [38].

Upon graduation, medical students enter a Foundation Programme, a two-year training program designed to give trainees a range of general experience before choosing an area of medicine in which to specialize. Subsequently they choose to train for a further three years to become a GP, or longer, to become a specialist consultant.

When a doctor has completed and gained the required skills and competencies, he or she is awarded a Certificate of Completion of Training (CCT), which allows registration on the Specialist or GP Register, a necessary requirement for practice as a consultant or general practitioner. Prior to registration, the GMC checks that the doctor in question holds the appropriate qualifications and has passed the necessary examinations [30].

### United States

The ABMS is a national, non-governmental, self-regulatory organization, comprising the 24 medical specialty member boards, and is the preeminent body responsible for the credentialing of medical specialists in the United States. Following receipt of a medical degree from an accredited US institution, candidates must undertake three to six years of training in an accredited residency and pass an examination in order to achieve initial specialty certification. The specialist evaluation is a rigorous process involving acknowledgement of educational requirements, professional peer evaluation and examination.

US specialty boards are jointly approved by the American Board of Medical Specialties (ABMS) and the American Medical Association Council on Medical Education (AMA/CME) [39].

### Summary-postgraduate training and specialist certification

A common finding in the five test cases is that medical education, and especially specialist training, is often self-regulated and entrusted in the hands of the profession. In Israel, the Netherlands and Germany specialist training is the direct responsibility of the NMA; the IMA Scientific Council regulates this process in Israel, the State Chambers of Physicians and the GMA hold the responsibility in Germany and the KNMG controls specialist training in the Netherlands. In the United Kingdom, the GMC, an independent body, took control of post-graduate training in 2010 and in the United States, the self-regulatory body of the American Board of Medical Specialists is responsible for the credentialing of medical specialists (Table 2).

**Table 2:** Specialty training

**Legislation**	**Responsibility**	**Assessments**	**Duration**	**Standards**
**GERMANY**				
State by-laws and independent statutes of the State Chambers of Physicians. GMA federal model training regulations.	Responsibility of the NMA (State chambers), accountable to the Governments of the Lander	Oral exam at the end of specialist training	4-6 years, depending on the specialty	Training and examination standards are set by the German State Chambers of Physicians (*Arztekammern*).
**ISRAEL**				
The Physician Ordinance and Physicians Regulations (Approval of Specialist Certification and Exams)	Responsibility of the IMA Scientific Council. Certification is formally issued by the Israeli Ministry of Health based on the recommendation of the IMA Scientific council.	Stage 1 written exam and stage 2 oral exam.	4-7 years, depending on the specialty	IMA Scientific Council holds statutory responsibility for training, examinations, and accreditation of departments for specialty training.
**NETHERLANDS**				
Individual Healthcare Professions Act (BIG Act).	Responsibility of the KNMG**.** The rules of the colleges (regulatory bodies of the KNMG) require approval of the Dutch Minister of Health	Each specialty has its own form of assessment, often administered throughout the training period.	2-6 years depending on the specialty	The KNMG determines the content of the training for medical specialists, accreditation of training institutes, and requirements for re-registration of medical specialists
**UK**				
The Medical Act (1983)	Responsibility of the GMC.	Physicians are required to complete examinations before being awarded a Certification of Completion of Training.	Post graduate education begins with a two-year Foundation training programme. Trainees then choose to train for an additional 3 year to become a GP or longer to become a specialist consultant.	The GMC sets the standards, learning objectives and requirements for postgraduate medical education in the UK.
**US**				
Most states have their own Medical Practice Act.	Responsibility of the American Board of Medical Specialties (ABMS)	Physicians must pass an examination in order to receive initial specialty certification	3-6 years of training	The ABMS is the preeminent body responsible for the credentialing of medical specialists. US specialty boards are jointly approved by the ABMS and the American Medical Association Council on Medical Education (AMA/CME).

### Physician disciplinary measures

Physicians are answerable to multiple layers of accountability, from professionally led regulation to hospital bylaws and regulation by health authorities. Professional self-regulation requires physicians to have an internal mechanism for receiving, investigating and dealing with complaints and allegations of professional misconduct or negligence.

Many health care experts believe that physicians, with their extensive educational background and medical knowledge following years of training and clinical experience, are best placed to judge their peers. However, it has also been argued that self-regulation can be self-serving and physicians may want to “protect their own”, and not appropriately reprimand their fellow physicians. For this reason, in many countries, medical disciplinary boards are composed of both medical professionals and lay persons. In almost all cases, public members make up a minority of the total membership of the regulatory bodies. The vast majority of representative medical associations support and encourage the concept of self-regulation of the medical profession [6].

### Germany

There are two possible disciplinary procedures in place in Germany - one under the professional code (*Berufsrechtliches Verfahren*) and the other under federal licensing law. In addition, in cases of treatment errors, patients can appeal to the arbitration board of the competent State Chamber of Physicians or sue a physician in civil court.

The GMA does not investigate or consider complaints. However, the professional codes of the State Chambers of Physicians are legally binding and define the rules of good conduct and duties of physicians. In case of an alleged breach of the professional code of conduct, the State Chamber may undertake procedures under the professional law.

In cases of minor breach of the professional code, the State Chamber of Physicians may issue a warning, which could also include a monetary fine, in accordance with the level of infringement. In the case of a serious breach, the Chambers may open proceedings at the official Court of Professional Conduct. If a physician is found guilty, there are several sanctions which can be applied, including a warning, formal reprimand or financial penalties. The most severe sanction the Chamber may issue is a statement of unreliability or unworthiness of the physician, which usually results in the withdrawal of his or her license [40].

Disciplinary procedures under federal licensing regulations fall under the competence of the government rather than the State Chambers of Physicians. The license to practice medicine is issued, and may therefore only be withdrawn, by the competent state health authorities of the federal states. If a physician has been found guilty of behavior that labels him unworthy or unreliable, his license will be revoked. The license may be suspended while a criminal investigation is ongoing and the physician will not be allowed to practise. Physicians may appeal against the decision to withdraw a license in the lower and higher administrative courts [23,40].

### Israel

Disciplinary measures for negligent, unethical or unlawful behavior in Israel fall under the auspices of the Ministry of Health. In addition, breaches of the IMA Ethical Code may subject a physician to sanctions by the IMA Ethics Committee, including a warning, rebuke or expulsion from the organization.

The Physicians’ Ordinance gives the Health Minister authority to suspend or cancel a physicians’ license, or alternatively, to issue a warning or censure, for various reasons, including serious medical incompetence or negligence. In addition, the National Health Insurance Law mandates the Minister to appoint an ombudsman to receive complaints from citizens on a range of administrative or clinical matters in the health system (Health plans and hospitals have individual ombudsmen) [34].

Besides the above, the Patients’ Rights Act of 1996 legislates the establishment of “examination committees” whose purpose is to examine specific adverse events that occur in medical institutions, including hospitals and ambulatory clinics. These committees can be convened by the head of the institution and/or the Director General of the Ministry of Health, upon receipt of a complaint by a patient or to look into an adverse event. The findings could potentially serve as the basis for disciplinary proceedings against a physician.

### The Netherlands

In the Netherlands, the BIG Act covers disciplinary rules for all health professionals. Disciplinary proceedings following complaints by patients or other parties are performed by independent judicial courts. These courts, known as *tuchtcolleges*, consist of two lawyers and three members of the medical profession; the latter are nominated by the KNMG [36]. The Central College in The Hague hears appeals and is made up of two medical and three legal professionals [28].

Various sanctions may be issued by the *tuchtcolleges*, including deletion from the BIG medical register; suspension from the register for a maximum of one year; probationary suspension of up to two years; restrictive entitlement to practice either for a limited period or for life; or a monetary fine [28].

In addition to the public disciplinary procedure, as contained in the BIG Act, there are also internal disciplinary procedures operated by the KNMG. The KNMG code of conduct provides guidance for physicians’ actions, noting the ethical and practical standards for physicians practice. Physicians may report their colleagues to the KNMG for breaches of the code.

According to the Rules of the KNMG disciplinary law, the Council may impose the following measures: warning; reprimand; denial or withdrawal to be a member of the board, commission or any other college within the KNMG for a period of up to five years; suspension of membership of a professional association; suspension of the individual KNMG membership for up to one year; deletion from the membership of a professional association or the individual KNMG membership [26].

### United Kingdom

In cases of alleged professional misconduct by physicians practicing in the UK, patients and medical professionals are advised to submit complaints to the GMC. Where serious misconduct is alleged, the GMC begins by investigating the case and deciding whether to refer it for adjudication. At the end of the investigation process, the case is considered by two case examiners (senior GMC staff), one medical and one non-medical, who have the authority to close the case without any action, issue a warning to the doctor or refer the case to a Fitness to Practice Panel.

At any time during the investigation, the GMC may refer the doctor to an Interim Orders Panel. This panel may suspend or restrict the doctor’s medical practice while the investigation is in progress.

The adjudication process consists of a hearing and referral to a Fitness to Practice panel.^d^ The Fitness to Practice panel hears the allegations against the doctor, determines whether they are substantiated and if so, what sanctions, if any, are appropriate. The panel is comprised of both medical and non-medical panelists appointed by the Council. For individual cases, a panel will usually consist of three to five members, one of whom must be a medical professional and one of whom must be non-medical, and a chairperson who may be medical or non-medical.

If the panel concludes that the doctor’s fitness to practice is impaired, it has the authority to impose any of the following: take no action, accept undertakings offered by the doctor provided the panel is satisfied that such undertakings protect patients and the wider public interest, place conditions on the doctor’s registration, suspend the doctor’s registration or erase the doctor’s name from the Medical Register, so that he/she can no longer practice medicine [30].

The BMA as a trade union may represent a physician in some types of disciplinary cases. However, they have no involvement in instituting disciplinary proceedings for physicians [41].

### United States

The individual states in the US are primarily responsible for dealing with complaints against physicians. Physician disciplinary procedures are often included in individual state medical acts. Following submission of a complaint, State Medical Boards must investigate the matter and, if necessary, evaluate a physician’s professional conduct and his ability to practice medicine [28]. When disciplinary sanctions are issued, the FSMB requires that the specific State Medical Board report these sanctions to a wide range of bodies, including the Federation, state and local medical societies, the AMA, the American Osteopathic Association and the National Practitioner Data Bank, as well as to certain government agencies [32].

Medical societies may discipline members for ethical violations, with the maximum sanction being expulsion from membership. Medical societies must also report breaches to the relevant government body or state board of medical examiners, providing any evidence of which they are aware regarding alleged criminal conduct of a physician in his or her medical practice. Any violation of governmental law may result in the physician’s being subject to civil or criminal liability [42].

### Summary-Physician disciplinary measures

In all but one of the case studies, the NMA is not involved in instituting disciplinary procedures for alleged physician misconduct. The exception is Germany, where there are two channels for disciplinary procedures: the professional code (*Berufsrechtliches Verfahren*), overseen by the State Chambers of Physicians, and federal licensing law.

In Israel, disciplinary proceedings are primarily under the jurisdiction of the Ministry of Health. In the Netherlands, they are performed by independent judicial courts; however, these courts ensure the representation of the medical profession and participation of the NMA and consist of two lawyers and three members of the medical profession nominated by the KNMG. The United Kingdom also ensures the participation of physicians in disciplinary procedures, as the GMC is the independent regulator of physicians and adjudicates complaints of professional misconduct. The BMA is not directly involved in this procedure, but may represent a physician in some cases. The AMA is also not officially involved in disciplinary measures, which are regulated and administered by the individual State Medical Boards in the US (Table 3).

**Table 3:** Disciplinary procedures

**Legislation**	**Responsibility**	**NMA involvement**	**Maximum sanction**	**Minimum sanction**	**Ethical breaches**
**GERMANY**					
Two possible disciplinary procedures: one under the professional code (*Berufsrechtliches Verfahren*) and the other under federal licensing law	Both the Government and the NMA	In case of an alleged breach of the professional code of conduct, the State Chamber may undertake procedures under professional law.	The Chamber of Physicians may issue a statement of unreliability or unworthiness, often resulting in competent state health authorities revoking the license to practice.	Warning	In case of an alleged breach of the professional code of conduct, the State Chamber may undertake procedures under the professional law.
**ISRAEL**					
The Physicians’ Ordinance	Government	The IMA is not responsible for disciplinary procedures; however they do issue certain sanctions for ethical breaches.	The Ministry of Health may suspend or cancel a physician’s license.	Warning	The IMA may issue sanctions for breaches of the IMA Ethical Code.
**NETHERLANDS**					
The BIG Act	Government	Disciplinary proceedings are performed by independent judicial courts. These courts (*tuchtcolleges)* consist of two lawyers and three members of the medical profession; the latter are nominated by the KNMG.	Deletion from the BIG registry and withdrawal of the basic medical registration.	Monetary fine	In the case of a breach of the KNMG code of conduct physicians may report their colleagues to the KNMG Council for Disciplinary Proceedings
**UK**					
The Medical Act (1983)	GMC	The BMA is not involved in instituting disciplinary proceedings for physicians, although they may represent them.	Suspension or removal from the medical register.	Warning	The GMC may issue warnings to physicians when their practice is not in-keeping with the GMC’s guidance on Good Medical Practice.
**US**					
State Medical Practice Acts	State medical board	When disciplinary sanctions are issued, State Medical Boards are required to report these sanctions to a wide range of bodies, including the AMA.	Disciplinary proceedings differ across states, however in many cases State Medical Boards may revoke or restore licenses.	Probation or license restrictions	Medical societies may discipline members for ethical standard violations by excluding them from the society. They must report any breaches to the relevant government body or state board of medical examiners

## Discussion

The findings indicate a wide variation in the extent of regulatory functions fulfilled by NMAs, in each of the three parameters discussed in this article. It appears that there are great differences among some of the countries studied, alongside similarities among others, as shown in Table 4.

**Table 4:** Summary

**Country**	**Mode of Governance**	**Licensing and registration**	**Specialty training**	**Disciplinary procedures**	**Score (Number of areas in which NMA is involved)**
**Germany**	Corporatist	Government-**NMA**	**NMA**	Government-**NMA**	3
**Israel**	Hybrid (with corporatist elements)	Government	**NMA**	Government	1
**Netherlands**	Hybrid (with corporatist elements)	Government	**NMA**	Government	1
**UK**	Entrenched command and control	GMC	GMC	GMC	0
**US**	Market	State medical board (Independent agency)	ABMS (Non-profit)	State medical board (Independent agency)	0

It seems that our findings strongly support all three hypotheses presented. In Germany, a classic representative of the corporatist model, the medical chambers assume the maximal number of regulatory tasks examined, more than any other NMA, and Germany therefore receives a score of three (hypothesis 1). The KNMG, which operates in a hybrid health care system that combines distinctive corporatist elements, assumes one task out of three (specialist training). As suggested in hypothesis 2, this is less involvement than that displayed by its German counterpart and more than that of the AMA and the BMA. Finally, both the AMA and BMA (market and entrenched command and control model respectively) assume no tasks at all, as suggested in hypothesis 3.

Interestingly, in all the countries discussed, regardless of the type of governance, specialty training is never under the direct control of the government, and, alternatively, there is significant involvement of the profession. The exact reasons for this are unclear, but may be related to the public desire for governmental supervision of the most “basic” level of professionalism, along with recognition of the expertise maintained by the profession, setting limits to governmental involvement in “higher” levels of medical professionalism.

In Germany, as in other Continental European countries such as Greece, Italy and Spain, a physician is required to join his or her NMA in order to practice medicine. In other countries, such as France and Austria, both registration and licensing is delegated to the NMA by the government. As far as we know, no European country has ever reversed the delegation of responsibility or taken it back into its own hands [22].

Professional self-regulation is the norm also in the Dutch health system [43]. In the Netherlands, regulatory functions are divided between the government and the KNMG, although the role of the KNMG is less significant than the role of the GMA in Germany since the KNMG has no standing as a chamber. It does, however, have several functions of a chamber, particularly in the area of residency training, where the NMA is responsible for the training of residents and the certification and registration of specialists [22,28]. The body responsible for these functions, known as the board of specialists, was founded in 1961 and since that time has been under the auspices of the KNMG, following a decade of debates between the KNMG and the government as to who would be responsible for such a body [43]. One must note that alongside a long history of self-regulation, the Dutch health system is also characterized by strong government regulation whose main purpose is to control health care costs (for example, strict monitoring of construction of new hospitals and the volume of their activities) [35].

We might have expected the Netherlands to resemble Germany more because of their geographical proximity and historical influences. After all, the Dutch health care system evolved originally as a Bismarckian system, similar to other European countries. A possible explanation for the difference in the standing of the KNMG and the German medical organizations may be traced to external historical developments.

Some have argued that the roots of modern representative medical associations date back to the formation of European guilds in the 12th and 13th centuries, and corporatism is merely a reincarnation of those guilds, with control over training, specialization, experience and therefore, the profession [6]. The Dutch guilds, for example, were dissolved in 1798 by the government established after the French Revolution. The KNMG was established about 50 years later, in 1849. Conversely, in large parts of Germany, the hold of the French was weak and therefore the German guilds continued to function undisturbed for a long period after their counterparts in the Netherlands were disbanded [10]. It is possible that this may at least partially explain why professional medical associations in Germany have a more “guild-like” character than the KNMG, and in their modern form even merited the status of a chamber.

In the United States market system, on the other hand, the business sector holds a prominent place in the national economy. This might affect, even indirectly, the greater involvement of private-public initiatives (usually by non-profit organizations) in the health system, and the collaboration between government and non-governmental organizations. Stacey argues that in the American system, as opposed to the German system, the State consciously distances itself from interference with the economy, and simultaneously distances other bodies, including professional associations, from similar intervention [8]. Therefore, essential medical regulatory activities are carried out by bodies such as the state medical boards, which on one hand function on behalf of the states, but which essentially have the standing of independent agencies within the health system of each individual state. Similarly, the ABMS is a non-profit organization unaffiliated with any governmental sector, but also not an integral part of the AMA.

It is also worth mentioning that US physicians have often viewed themselves as solo practitioners, not part of any collective association. We can assume that this strong sense of personal freedom possessed by American doctors combined with the nature of the American economy as a private business oriented system have limited the role of the AMA as a regulatory body.

In the UK, where the government is responsible for the provision and financing of health care services through the NHS, the regulatory model differs from that in the US or Germany. The BMA influences health policy in Britain, and is regarded by the government as a serious partner in decision making. Nonetheless, the BMA is defined as a trade union rather than a chamber.^e^ Although the Association represents physicians and promotes their interests, the responsibility for regulatory functions is delegated by the government to the GMC, the body statutorily responsible for regulation and quality assurance in medicine. The GMC, founded in 1858, represents a long tradition of state-sanctioned professional-led regulation in the UK. This arrangement served both the medical profession and the state in the frame of a socio-political bargain; the physicians gained professional autonomy (albeit not complete) in exchange for their obligation to act in the interest of the public. Whereas the BMA’s prime purpose is to serve the interest of the profession, the GMC clearly stems from governmental decisions and statute [11,44]. Moreover, in recent years it seems that the state has strengthened its control over the GMC, increasing the number of lay members in the Council [45].

It seems that the connection between the involvement of NMAs in regulatory tasks and their assumption of the duty of collective bargaining is a complex one and it is hard to indicate a coherent pattern between them. We believe that this issue deserves further exploration beyond the scope of this paper.

It is interesting to dwell for a moment on the similarities and differences between the UK and Germany regarding the relationship between the government and the profession. In both cases we see that physicians have a certain amount of autonomy, but this autonomy was achieved, and is currently regulated, differently in the two countries. The British model was shaped through efforts of doctors to preserve their autonomy and stave off government regulation. This is evident in the continuing struggle between physicians and the government for dominance over the GMC. In Germany, too, we see a system of self-regulation, but one with characteristics different from those in Britain, one that sports closer collaboration with the government through broad powers on the part of the medical chambers in the various districts. These chambers tend to intervene in the professional life of the doctor and instituted a detailed system of ethical and professional codes, some of which are included in federal statutes [18].

Whereas self-regulation in Germany means widespread collaboration between the doctors and the government in a variety of regulatory activities, self-regulation in Britain was used as a strategy to create a separate world of governance, closed to the wider audience of the government or the free market. The contrast between the British and the German systems, according to Moran, can be seen in the characteristics of the GMC. The GMC plays a minor role in the health system when it comes to financing and provision of services, and has a narrow self-definition regarding its institutional functions, and consequently, its resources are similarly modest. As a result one can say that the “social contract” between the government and the profession in Germany has greater importance, even legally, than it does in Britain [18].

From this point of view it is possible to gain perspective on the influence of economic, political and bureaucratic circumstances on the division of labor between NMAs and the government through institutional arrangements shaped and developed throughout history.

### What about Israel?

Israel is absent from many of the different typologies conducted in the field of health policy over the years. It is difficult to assign a specific paradigm to the country. Those who would assign it a corporatist model point to the organization of the health system as one based on insurance coverage by the sick funds. Those who see it as more of a state system point to the National Health Insurance Law, with its universal character and government regulation of health services. Finally, the steady rise in private expenditures on health, which is one of the highest among OECD countries, brings it closer to a market system, although at this stage this is not the dominant mode of governance.

Nonetheless, when it comes to responsibility for regulatory mechanisms, we see a great similarity between Israel and the Netherlands. Along with Germany, responsibility is divided in all three countries between the government and the NMA. Over the years, this model became fixed as the natural and almost self-evident working model in Israel, although from time to time disagreements would (and still do) arise between the two bodies as to existing boundaries. However, when studying the different international models, it is not at all self-evident why Israel is so similar to those countries based on a corporatist model. Why did Israel not develop a body such as the British GMC, or independent or semi-independent agencies such as in the American model? After all, both the UK and the US always had and continue to have great influence on the political and economic systems in Israel. In order to begin to shed some light on this question, we must understand the roots of the Israeli health care system, as well as how the regulatory model developed to that which is familiar to us today.

The Israeli health care system began developing many years before the founding of the State, and therefore the associations that sprung forth from the medical community preceded national institutions in shaping its character. Already at the start of the twentieth century, thousands of immigrants were treated by a small group of physicians, themselves immigrants from Central and Eastern Europe. In 1909, these physicians numbered a mere 27, and were not connected to any political party or stream [46]. They performed their work individually, rather than as part of organizations or medical institutions. However, this pattern of individual care did not last long, and beginning in 1911 a number of “sick funds” were established. These provided members with basic health services, both primary care and hospitalization, for a fixed membership fee, and served as an early model of the corporatist system. The Israeli Medical Association (then known as Hebrew Medicinal Society for Jaffa and the Jaffa District, later as The Hebrew Medical Association in the Land of Israel) was founded shortly thereafter, in 1912.^f^

In May 1948, the State of Israel was established and with it, the Ministry of Health, which inherited an already existing and operating health care system. During these years, the medical profession operated under a collectivist and hegemonic model that characterized the first few decades of the State. This model of “building the nation” was first and foremost characterized by having the State as the central source of authority in the establishment of dominant social institutions.

In the context of this hegemonic model, professional groups, among them physicians, did not attain significant social power [47].^g^

Against this backdrop of historical, political and social circumstances, the IMA’s request to be considered a chamber was rejected. Even before the ink dried on the Israeli Declaration of Independence, on June 1, 1948, the IMA’s Central Committee sent a memorandum to the Minister of Justice requesting status as a chamber, meaning that all doctors would be statutorily required to have membership in the Association. As previously noted, the idea of a chamber is not an Israeli invention. German doctors are members of chambers, and this arrangement is the norm in other European countries as well. The defining characteristic of chambers is their statutory authority; similarly, the IMA wanted all doctors to join its rank by law, and the organization, in turn, would maintain high standards of medical and professional ethics, grant and suspend, where necessary, medical licenses, and grant specialty certification. The government, however, rejected the IMA’s application and did not grant the hoped for monopoly [48]. The result was that from then until today the IMA has no real enforcement power or licensing authority over its members. The initial vision of nationalism that reigned in the early years of the State pushed aside the medical profession in all things connected to the regulation of the health system and sought to concentrate authority in the hands of government institutions.

In this context, it is interesting to compare Israel and Germany, since a similar process occurred in both, with opposite outcomes. In 1874, three years after the German states were combined into the German Empire, the medical associations approached the governing bodies of the fledgling Empire with a request to establish chambers, membership in which would be mandatory according to federal law. Such chambers already existed in several states before the unification, including Baden, Bavaria and Saxonia, but the doctors sought official recognition from the Empire. To their disappointment, Chancellor Bismarck held that the Reich was not the appropriate framework for establishing such regulation. Despite the lack of federal legislation, in 1878 the profession organized a system of state level chambers as mandatory physicians’ organizations alongside voluntary scientific associations. A similar process occurred in most of the German states, which adopted such a policy as a solution to the increasing need for professional discipline among physicians. Although ethical codes existed in the scientific associations, membership in the associations was voluntary and so subscription to the codes could not be optimally guaranteed. The doctors, for their part, saw the establishment of chambers as a means of preventing unfair competition by those practicing without a license [49]. In sum, the German medical associations achieved and preserved the standing of chambers, whereas the IMA did not. This may explain the difference in the level of regulatory involvement between them. At the same time, a similar explanation may serve to clarify the striking similarity between IMA and the KNMG, both of which operate in a hybrid-corporatist system while lacking the formal standing of a chamber, leaving them with only very limited regulatory authority.

Nonetheless, the government hegemony in Israel is not complete. Control over residency and certification was given to the Scientific Council, which is part of IMA. In light of the events described heretofore, one can ask why the governmental hegemony in the area of medical regulation was never completed, and why postgraduate training remained in the hands of the profession.

There is no doubt that satisfactorily answering this question requires in depth historical research; nonetheless, it may well be that the influence of German immigrants played a part in this development, at least to some degree. In 1936, the central scientific committee for the organization of scientific lectures was established. From these modest beginnings developed the Scientific Council of the IMA, with its attendant broad set of powers [50]. This occurred at the height of the 5th wave of Jewish immigration to Palestine from Central Europe, known as the “German *aliya”* (immigration), which was enormously significant in terms of its influence on medicine in Israel. The Central European Jewish immigrant physicians brought about a true upheaval in the area of medicine as a result of the vast differences in the quality of medicine, the type of organization and the professional values they brought with them, as compared to earlier immigrants from Eastern Europe [46].^h^ From a numerical perspective, Germany was the country of origin of the largest group of immigrant physicians - 35% of the total number of physicians. Furthermore, Germany was the country in which most physicians (68%) at the time studied. By comparison, the next country in terms of number of medical students was Russia, in which only 19% of the immigrant doctors studied [51].

One of the major differences between the German-Jewish immigrants and their older colleagues was the former group’s tendency to specialize in specific fields of medicine, as compared to their predecessors who tended more to be general practitioners. The trend of specialization spread greatly as a result of the arrival of the German-Jewish immigrants, and changed the face of medicine in Israel, particularly in certain fields such as surgery, X-rays and psychiatry [46]. It may be that the scientific dominance of German-Jewish physicians, their predilection for specialization and their attempts to assimilate into the local system the organization and working procedures to which they were accustomed in their country of origin, partially contributed to the establishment of the Scientific Council under the auspices of the IMA.^i^

The Scientific Council is responsible for the post-graduate training and specialization of doctors by law. Thus, in effect, the IMA did end up with some statutory powers (those dealing with specialty certification), similar to the European medical associations, and especially the KNMG.

It is interesting to note that, despite the current situation, retention of specialty certification by the profession is not self-evident. An attempt was made by the government in 2009-2010 to move the Scientific Council from the IMA’s jurisdiction to that of the Ministry of Health. In response, the IMA launched a campaign aimed at convincing government officials and members of Knesset of the importance of leaving this responsibility in the hands of the profession. This campaign was successful in thwarting such efforts at least to this point.

## Conclusion

The regulation of the medical profession differs throughout the world. In Continental Europe, co-operation between the NMA and the government is more common. This kind of regulatory model reflects the corporatist political-economic system historically rooted in countries such as Germany, and at least to some extent in the Netherlands. Conversely, in the US, where the private sector is more prominent, the state consciously distances itself from interference with the economy, and simultaneously distances other bodies, including professional associations, from similar intervention.

Accordingly, the AMA does not itself assume regulatory functions, leaving these for independent non-governmental bodies in the health care system. In the UK, the BMA is defined as a trade union and the GMC fills the regulatory missions delegated to it by the government.

Considering the far-reaching American influence on Israeli economy and politics and the long period of British rule in Palestine, the current European-like regulatory model in Israeli medicine is not self-evident. As we have suggested, for historical and ideological reasons, the Israeli health care system evolved initially as a corporatist German-like model. With the establishment of the State of Israel, this model encountered a strong social-political regime that aspired to impose a statist regulatory approach and exclude professional elites from holding powers of strategy, economy and administration. This may explain the state’s refusal to grant IMA the standing of a chamber, as opposed to the standing of the German physician organizations. It may also explain the striking similarity between IMA and the KNMG – both organizations operate in a hybrid-corporatist system while lacking the formal standing of a chamber, leaving them with only limited regulatory authority.

Finally, as we mentioned earlier, context and culture are deciding factors in the development of health systems. The Israeli case, as well as the others, illustrates the potential roles of history and ideology in shaping contemporary regulatory models. We would like to see this issue further explored through historical and comparative research, with the hope of finding additional explanations for the regulatory path that has evolved in Israel.

## Endnotes

^a^In certain countries, some form of recertification/revalidation is required after a fixed number of years. There are wide differences in the recertification schemes. In some cases doctors may be required to present a notice of ‘good standing’, stating that no complaints have been issued against them; in others, they must aver that they have taken part in Continued Medical Education (CME) and/or Continued Professional Development (CPD).

^b^Chambers are self-governing statutory bodies that represent the interests of their members, monitor compliance to professional standards and are responsible for specialty training and continuing medical education. However, the chamber does not actually grant or suspend medical licenses.

^c^Students from specific countries recognized by the Ministry of Health are exempt from the licensing exam. The Ministry is currently looking into expanding this list of exemptions in order to combat the physician shortage.

^d^The Fitness to Practice procedure is outlined in the 1983 Medical Act and the Fitness to Practice Rules of 2004.

^e^On one hand, the BMA and the AMA are similar in their lack of participation in regulatory activity. On the other hand, the BMA differs distinctly from the AMA, in that it is also a trade union. In that respect the BMA somewhat resembles its European counterparts, representing its members in collective bargaining and negotiating wages and work conditions (although in Germany, the chambers generally do not engage in collective bargaining themselves, but concentrate on their regulatory missions, leaving wage bargaining in the hands of other corporatist bodies - the regional statutory health insurance physician associations) [20].

^f^Further historical background can be found in Appendix.

^g^This observation nonetheless raises an interesting question, namely: how is it that the Israeli Bar Association (IBA) managed to secure such broad autonomy for its members? The Israeli Bar Association Law was first enacted in 1961 and took effect in 1962, 18 years after the first draft and 14 years after the establishment of the State, and following countless obstacles and reservations. It is a precedent setting law and the first of its kind in Israel. According to Dr. Yehoshua Rottenstreich, the first chairman of the IBA, the law was the result of a political coup, good connections and no small amount of luck. Support for the Bar Association Law among Knesset members was achieved in exchange for legal assistance with the defeat of a different bill, The Inheritance Law, which did not sit well with many of the members [52]. One can say that the establishment of the IBA as an independent statutory entity was achieved in spite of the government’s position, and not because of it, and in any event does not represent the general approach of the time towards groups perceived as “social elitists”.

^h^Note that despite the name given to this aliya, immigrants from Germany, Austria and Czechoslovakia represented only one fourth of all immigrants between the years 1933-1939, and numbered between 50,000-60,000 persons [53].

^i^Nonetheless, we must not exaggerate the power of their immediate impact, keeping in mind that German-Jewish physicians were newly arrived immigrants from a completely different geographical and cultural region, struggling against difficult conditions. Many of them weren’t employed at first, as the market was flooded with specialists in a very short period of time [54].

## **Appendix-Historical background to the development of medicine in Israel**

In 1909, there were only 27 physicians in what was then Palestine, and these were not connected to any political party, organizations or institutions, but rather performed their work individually. In 1911, the Workers Health Fund Kupat Holim Clalit (today the Clalit Health Services) was founded, and the Israeli Medical Association (then known as Hebrew Medicinal Society for Jaffa and the Jaffa District, later as The Hebrew Medical Association in the Land of Israel) was founded shortly thereafter, in 1912. Between the years 1911-1915 a number of sick funds were established in Palestine by socialist-imbued Jewish pioneers working as day laborers in Jewish agricultural settlements. The funds provided members with basic health services, both primary care and hospitalization, for a fixed membership fee. In 1921 the sick funds amalgamated and began operating under the auspices of the Jewish Labour Federation (Histadruth), a trade union framework founded a year earlier, in 1920. Kupat Holim became a Labour Federation-owned entity, controlled and operated under Labour Federation management, and designed to serve the health needs of trade union members and their families [55].

The collective organization of the health system at the turn of the century expressed not only the immediate need for health services, in light of serious diseases such as malaria, but also the ideology of the socialist pioneers that rejected any professional model that was not collective. In this way, common ground was found between the objective needs and the socialist world view of the Zionist establishment in the Jewish community in Palestine during the British rule. The health system in the Palestine Jewish community of the 1920's was built from scratch by two organizations: one philanthropic, the American Zionist Women Organization "Hadassah", and the second voluntary, Kupat Holim. Most of the immigrant physicians were absorbed by these two organizations and employed under collective work agreements, largely preempting the need for the establishment of a private medical infrastructure [46,56].

Clearly, the evolving health care system of the Jewish community in Palestine took the form of a corporatist model at that stage, providing essential medical services and fulfilling the collective vision of the pioneers. This to some extent explains the difference between the regulatory model in Israel and that in the US. It also sheds light on the difference between the Israeli model and its British counterpart, despite the lengthy period of the British rule in Israel and its wide influence on the Israeli legal system.In May 1948, the State of Israel was established and the newly formed Ministry of Health inherited an already existing and operating health care system. During these years, the medical profession operated under a collectivist and hegemonic model that characterized the first few decades of the State. This model of "building the nation" was first and foremost characterized by having the State as the central source of authority in the establishment of dominant social institutions. The economic, medical, legal and military systems and even the individual saw themselves as recruits of the national, collective goals and were subordinate to them [47].

 Then Prime Minister David Ben Gurion tried to impose a governmental model, successfully implemented in the military and educational systems, on the already operating health system, but this idea was rejected by the Histadruth Labor Union, which held great political power In point of fact, health insurance coverage remained in the hands of public, non-governmental institutions, due to a series of differences that arose over the years between the Ministry of Health and the Clalit sick fund [57].

## Competing interests

The authors declare they have no competing interests.

## Authors’ contribution

The authors jointly conceived of the concept and design of the paper. MG focused primarily on data collection and drafting the empirical sections of the paper. BL focused primarily on the methodological, historical and theoretical sections of the paper. MB was primarily responsible for data integration and editing. All authors read and approved the final manuscript.

## Authors’ information

Malke Borow is the director of the Division of Law and Policy at the Israeli Medical Association, which covers the fields of law, health policy and international relations. She holds a BA in psychology from Queens College/CUNY and a JD from Columbia University in New York.

Baruch Levi is a researcher of health policy at the Israeli Medical Association. He holds a BA in Economics and International Relations from the Hebrew University in Jerusalem, an MA in Political Science from Tel Aviv University and is currently working on his PhD about the effects of economic policies on public health.

Michelle Glekin is an International Relations Officer at the Israeli Medical Association. She holds an MA in psychology from the University of Edinburgh.
